# Testing the morphological constraint hypothesis of tail length in the sexually dimorphic *Cerastes vipera* and new perspectives

**DOI:** 10.1038/s41598-023-31624-6

**Published:** 2023-03-17

**Authors:** Jaim Sivan, Itay Tesler, Shlomo Hadad, Abraham Allan Degen, Eli Geffen, Michael Kam

**Affiliations:** 1 Department of Life Sciences, Achva Academic College, M.P. Shikmim, 79800 Arugot, Israel; 2 Department of Life Sciences, Ben Gurion University of the Negev, 8410500 Beer Sheva, Israel; 3 Negev Zoo, P.O.B. 4033, Beer Sheva, Israel; 4 Desert Animal Adaptations and Husbandry, Wyler Department of Dryland Agriculture, The Jacob Blaustein Institutes for Desert Research, Ben Gurion University of the Negev, 8410500 Beer Sheva, Israel; 5 School of Zoology, Tel Aviv University, 69978 Tel Aviv, Israel

**Keywords:** Zoology, Herpetology

## Abstract

The morphological constraint hypothesis (MCH) states that, in snakes, males typically have relatively longer tails than females to accommodate the hemipenes and retractor muscles. To date, most studies testing the MCH have been interspecific and results have been equivocal. We tested the MCH intraspecifically on *Cerastes vipera,* a species with a relatively short tail and suitable for testing the MCH. The relative tail length and length of the hemipenes pocket in *Cerastes vipera* were measured in preserved museum-maintained males (n = 35) and in free-ranging males and females (n = 277). Males exhibited relatively longer tails than females, which was explained fully by the length of the hemipenes pocket. The relatively short tail of *C. vipera* presents a constraint to the reproductive structures in males, as the length of the hemipenes pocket occupies a greater proportion in shorter- than longer-tailed individuals. This is the first report presenting these intraspecific findings in support of the MCH. Whether these relations are widespread among snake families, within Viperidae, or specifically within *C. vipera* warrants further studies.

## Introduction

In numerous snake taxa, including Colubridae^1^ and Viperidae^2–4^, males typically have relatively longer tails than females when the comparison is based on body length^2^. Reasons for this difference in tail length and sexual dimorphism within species has led to three major hypotheses by King^5^:

1. “The morphological constraint hypothesis proposes that males have relatively longer tails than females to accommodate the hemipenes and retractor muscles” ^5^. This hypothesis was first presented by Klauber^2^ and Clark^1^ and implies that tail length dimorphism is explained by the length of the reproductive structure.
2. The female reproductive output hypothesis, first proposed by Kaufman and Gibbons^6^ and Semlitsch and Gibbons^7^, states that “females have relatively shorter tails than males as a secondary result of natural selection for increased reproductive capacity”^5^. That is, the shorter tail length corresponds to larger body cavities in females.
3. The male mating ability hypothesis, also first proposed by Kaufman and Gibbons^6^ and Semlitsch and Gibbons^7^, states that “sexual selection favors relatively longer tails in males during courtship”^5^.

These hypotheses have been tested mainly interspecifically, and within Colubridae, and the morphological constraint and the female reproductive output hypotheses^5^ received some support. However, as natural selection works on successful qualities within species^8,9^, an intraspecific analysis is warranted. To date, results have been equivocal. The male mating ability hypothesis was supported recently in a species displaying male-to-male combat during courtship^10^. The morphological constraint hypothesis was not supported in an early intraspecific study on the water snake, *Nerodia sipedon*^5^, but was supported partially in a later study on *Thamnophis sirtalis parietalis*^11^, both Colubridae species. In this latter study, the hemipenes length explained less than 10% of the variance in relative tail length. However, it should be noted that relative TL in that study was tested against absolute hemipenes length, whereas the proportion of the reproductive structure to TL is presented in the original hypothesis. To date, this hypothesis has been tested only on Colubridae species.

Based on the morphological constraint hypothesis, the hemipenes and the retractor muscles pose a greater constraint on minimum tail length (TL) in shorter-tailed snake species, and, therefore, their TL should be more male biased^5^. Thus, the morphological constraint hypothesis should be best tested intraspecifically in species with a relatively short tail and without displaying male-to-male combat during courtship.

Given these criteria, *Cerastes vipera*, a small desert dwelling Viperidae^16^, is suitable to test the morphological constraint hypothesis. In the present study, the subcaudal number of scales and the length of the hemipenes pocket were used as a measure of the hemipenes constraint size and was tested against a relative measure of TL. We tested whether the hemipenes pocket can explain the longer tail in males, and predicted that males with more subcaudals or with a longer hemipenes pocket would possess relatively longer tails. Furthermore, our design allowed for testing King’s prediction^5^ intraspecifically, that is, that the male reproductive structure occupies a greater proportion of TL in short than long-tailed individuals.

## Methods

### Study species

The Saharan sand viper, *Cerastes vipera* (Linnaeus 1758), is a small fossorial viperid. It is a sidewinding, mainly nocturnally-active species, and its distribution is limited to sand dunes across northern Africa deserts. In Israel, it inhabits the western Negev. It preys on small lizards, predominantly the Nidua fringe-fingered lizard and *Acanthodactylus scutellatus*^12,13^, using sit-and-wait ambush as the main hunting strategy, and venomous fangs. It remains in a coiled position under the top layer of sand with only eyes and nostrils protruding slightly^13,14^. *C. vipera* uses body oscillating movements to burrow into the sand. In addition, *C. vipera* shows sexual dichromatisation where the tip of the female tail is black, whereas that of the male is not, which enables clear differentiation between sexes^13,15^.

### Sexual dimorphism in tail length

Sexual dimorphism in tail length, where males have relatively longer tails than females, is a common occurrence among snake species^1,2,5^. It was assumed to be valid in *Cerastes vipera* as well, but has not been tested. Sexual dimorphism in tail length was determined based on a general linear factorial model (GLM; ANCOVA design). Tail length was set as the dependent variable, sex as a factor, and SVL as the covariate. To compare male and female slopes we included the interaction between sex and SVL. A significant interaction implies a different tail—SVL ratio between sexes. In the present study, we used our large data base on free-ranging *C. vipera* collected over 15-years, in which snout-vent-length (SVL) was published earlier^16^. Snake tracks were followed and then the snakes were captured by hand, stretched gently on a solid board and measured for SVL and TL to the nearest 0.5 mm using a 30 cm solid ruler. To minimize errors in SVL due to the person doing the measurements^17^, two researchers (JS and SH) made all measurements in this study. All procedures on the snakes were in accordance with the guidelines set by the Animal and Nature Reserves of Israel and were approved by the Ben-Gurion University Committee for the Ethical Care and Use of Animals in Experiments (Authorization Number IL-60-11-2018-B). Tail length (TL) is published here for the first time (Appendix 1). Data include juveniles and adults, but not neonates. To compare data from this study with data from the literature, sexual dimorphism in TL (TLD) was determined based on the difference in the residuals of TL on SVL between males and females following King^5^. To allow for comparison of the sexual dimorphism in TL in families other than Colubridae, we also calculated the “coefficient of divergence” (CD) that was defined by Klauber^2^ as the difference of average TL/total body length in males minus average TL/total body length in females as the percentage of average TL/total body length in males and females.

### Hemipenis pocket measurements

We used preserved *C. vipera* males (Ben-Gurion University Zoological Museum), which were collected at the same site where measurements were made on free-ranging *C. vipera*^9^, to determine the length of the hemipenis cavity. The length of the left hemipenes pocket (HP) was measured to ± 0.01 mm using a 1.68 mm wide probe and a caliper (MRC, sensitivity ± 0.01 mm), while the number of sub-caudals covering the hemipenes pocket was counted concomitantly. For measurements of SVL and TL, snakes were stretched gently on a solid board and SVL and TL were measured to ± 0.5 mm using a 30 cm solid ruler^16^. The data are based on earlier measurements in free-ranging *C. vipera* that were done with permission from the Israel Nature and National Parks Protection Authority^16^. All data are presented in Appendix 2.

### Testing the morphological constraint hypothesis

We calculated tail length with and without the hemipenes pocket (thereafter tail state), and compared these relative tail lengths using GLM. In the GLM model, tail state was set as the factor and SVL as the covariate, and the interaction between tail state and SVL was used for testing whether there was a difference in the relative tail length. As the variance for TL in the museum specimens was relatively greater than that of free-living snakes, we also used the robust regression model to accommodate for outliers. For comparison with the methodology taken in past studies, and the fact that the length of the hemipenes pocket in terms of number of subcaudals does not change during growth, we tested the effect of HP on tail length using the residuals of tail length on SVL.

Following the hypotheses of King^5^, Shine et al.^11^ suggested to test the morphological constraint hypothesis intraspecifically using the residuals of relative TL on the size of a “male reproductive structure”. For comparison alone, we also employed a similar analysis using the hemipenes pocket as a “male reproductive structure” and following King’s prediction (Prediction 1B^5^) based on the morphological constraint hypothesis, we tested the effect of relative TL (the residuals of TL on SVL) on the ratio of the hemipenes pocket length and TL.

### Statistical methods

To compare between sexes, we used General Linear Models (ANCOVA design). In addition, linear regression and robust regression models were used to calculate slopes within groups. Data were analyzed using JMP Pro (version 17, SAS Inc.). For ratio comparisons with past studies, the TL:SVL ratios were logit transformed to account for the bounded nature of the response^19^ and were tested statistically for difference between groups using a two-tailed t-test. P < 0.05 was accepted as the level of significance and values are presented as means ± SE.

## Results and discussion

### Sexual dimorphism of tail length in *Cerastes vipera*

**Figure 1:**
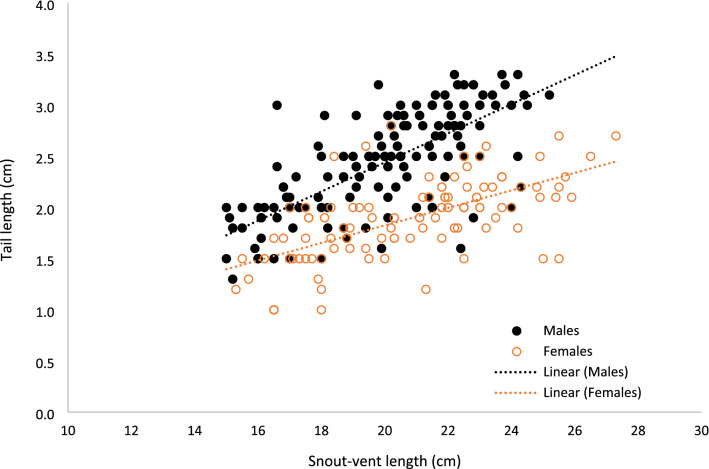
Tail length vs snout-vent length in free-ranging *Cerastes vipera*, illustrating relatively longer tails in males than in females. See text for statistical tests.

TL and SVL in free-ranging *C. vipera* were 2.4 ± 0.34 and 19.9 ± 0.23 in males, and 1.9 ± 0.03 and 20.8 ± 0.27 in females, respectively (Table 1). Overall, SVL explained 24% of the variance in TL of free-ranging *C. vipera* (r^2^ = 0.24, F = 86.3, *p* < 0.0001, n = 277; Fig. 1). A General Linear Model (ANCOVA design) explained 60% of the variance in TL, and indicated that TL in males is relatively longer and the slopes of the regression of TL on SVL differed significantly between sexes, and was greater for males (Table 2). Regression analysis for each group separately yielded significant (*P* < 0.0001) slopes of TL on SVL (0.142 ± 0.01 and 0.086 ± 0.010, in males (r^2^ = 0.51) and females (r^2^ = 0.40), respectively. Similar slopes for sexes were also calculated using robust regression models (slopes 0.149 ± 0.011 and 0.088 ± 0.010 in males and females, respectively). Consequently, our initial hypothesis was supported strongly as *C. vipera* males possess relatively longer tails than females. When examining each sex separately, SVL explained a greater part of the variance in TL for each sex (r^2^ = 0.39; n = 111 in females; r^2^ = 0.51; n = 166 in males). Interestingly, a similar analysis of another small viperid, *Bitis schniederi*, found no difference in the slope of the regression of TL on SVL between sexes, although their ANCOVA yielded significant sexual dimorphism, with males having relatively longer tails than females^20^.

**Table 1:** Average total length (L), snout-vent length (SVL), tail length (TL) and ratios, in free-ranging *Cerastes vipera* males and females. Included are preserved male specimens (Males*) in which subcaudal scales covering the hemipenes pocket (SC) and the length of the hemipenes pocket (HP) were determined.

	Males	Females	Males* (full tail)	Males* (full tail—HP)
N	166	111	35	35
Total length (cm)	22.3 ± 0.22	22.7 ± 0.30	24.0 ± 0.35	24.0 ± 0.35
SVL (cm)	19.9 ± 0.23	20.8 ± 0.27	21.2 ± 0.30	21.2 ± 0.30
TL (cm)	2.4 ± 0.34	1.9 ± 0.03	2.76 ± 0.080	2.01 ± 0.073
SC			6.8 ± 0.21	
HP (cm)			0.75 ± 0.030	
TL/L	0.108 ± 0.001	0.083 ± 0.001	0.115 ± 0.003	
TL/SVL	0.122 ± 0.001	0.091 ± 0.001	0.130 ± 0.003	0.095 ± 0.003
SCL/TL			0.276 ± 0.011	
r^2^	0.513	0.395	0.197	0.106
Linear regression	0.14 ± 0.01	0.08 ± 0.01	0.12 ± 0.04	0.08 ± 0.04
Robust regression	0.15 ± 0.01	0.09 ± 0.01	0.12 ± 0.05	0.08 ± 0.04

Linear and robust regression slopes and r^2^ of tail on SVL are presented. Values are means ± SE. See text for details.

**Table 2:** General linear model estimates and likelihood-ratio effect tests for free-living and preserved *Cerastes vipera*.

Term	Estimate (± SE)	L–R χ_1_^2^	*P*
Free-living (n = 277)			
Sex (F)	−0.316 ± 0.020	173.9	** < 0.001**
SVL	0.114 ± 0.007	169.0	** < 0.001**
Sex(F) * SVL	2.4 ± 0.34	14.1	** < 0.001**
Preserved specimens (n = 70)			
Tail state (Full tail)	0.376 ± 0.049	42.7	** < 0.001**
SVL	0.097 ± 0.028	11.4	** < 0.001**
Tail state (Full tail) * SVL	0.020 ± 0.028	0.5	0.482

Significant effects are denoted in bold.

The average TL:SVL ratio was greater (t = 14.57, df = 288, *p* < 0.0001) in males than in females (0.122 ± 0.001 vs 0.091 ± 0.001). Average residuals of the regression of TL on SVL in males and females were 0.248 and -0.372, respectively, and, TL difference (TLD) was 0.620. This value is much greater than all other known TLDs reported by King (range -0.12 to 0.14 in full dataset in genera of Colubridae snakes), who employed the same method of calculations. To compare *C. vipera* with Klauber’s data^2^, we also calculated TL as a proportion of total length, which was 0.108 ± 0.001 in males and 0.083 ± 0.001 in females. Hence, the coefficient of divergence in TL (CD) in *C. vipera* was 26.0%, which falls among the highest 30% values calculated similarly for 48 snake species belonging to a number of families, including Colubridae, Boidae, Lamprophiidae, Elapidae and Viperidae. *Cerastes vipera* also had the highest CD of the four Viperidae genera, namely *Agkistrodon*, *Atheris*, *Bothrops* and *Trimerusurus*^2^, in that study. It should be noted, however, that *Atheris*, *Bothrops* and *Trimerusurus* are arboreal or semi-arboreal, and, therefore, have long tails associated with their ecology, and which may explain, at least in part, their relatively low CDs.

A high CD in species with relatively shorter tails was mentioned by Clark^1^ but from a different perspective. Based on the view that “the tail tip is used as an anchor against which the snake can push and move itself forward … An elongated tail, however, would tend to bend readily when pushed against while a shorter tail would be stronger and better suited”, he suggested that the “increased divergence in length of tail between the sexes and shortening of the tail are associated with burrowing habits”. This possibility has been hypothesized for Colubridae and could be considered for *C. vipera* due to its burrowing habit and hunting strategy where the relatively short tail forms a basis for striking. To effectively strike a potential prey, the small-sized snake would require a “stronger and a better suited” relatively short tail. A similar idea but from a different perspective was raised by Lillywhite et al.^21^ who suggested that Viperidae snakes may keep digesta in the digestive tract for a longer period to be used as a better and heavier base for striking, which is specifically important for small species.

### Testing the morphological constraint hypothesis of tail length in *Cerastes vipera*

Mean (± SE) body mass (m), snout-vent length (SVL) and tail length (TL) of preserved male *Cerastes vipera* (n = 35) were 15.6 ± 0.51 g, 21.2 ± 0.30 cm and 2.76 ± 0.080 cm, respectively (Table 1). SVL explained 20% of the variation in TL in these males (r^2^ = 0.197, F = 8.08, p = 0.0076, n = 35), which was similar to that of the free-ranging *C. vipera*. A General Linear Model (ANCOVA design) explained 51% of the variance in TL in the preserved specimens (where both full TL and TL-HP were used) and indicated a significant effect of tail state although slopes did not differ (Table 2). However, the slopes of the linear regression of TL on SVL for full TL and TL-HP of 0.12 ± 0.04 and 0.08 ± 0.04 were similar to those in free-living males and females (Table 1). Similar slopes were evident when using robust regression models, that is, 0.12 ± 0.05 and 0.08 ± 0.04 (Table 1). The TL:SVL ratio was 0.130 ± 0.0035, which differed slightly (6.3%), although significantly, from the ratio of 0.122 ± 0.001 in free-living males (t = -2.39, df = 199, *p* = 0.018), but was substantially greater (35.5%) than the ratio of 0.091 ± 0.001 in free-living females (t = −11.12, df = 144, *p* < 0.0001) (Table 1). Differences in average morphometric measurements between museum males and free-living males and the relatively greater variance in the regression models of the museum males might be due to the large difference in sample sizes and/or in measuring preserved specimens vs living ones. However, and as noted above, slopes of the regression analyses of free-living males and preserved males were similar as were the slopes for free-living females and the preserved males when tail length was taken as TL-HP.

The number of subcaudal scales covering the hemipenes pocket in males averaged 6.8 ± 0.20 (range 4 to 9). The length of the hemipenes pocket averaged 7.51 ± 0.299 mm (range 4.27 to 11.22 mm), accounting for 27.6 ± 1.09% of TL (range 14.9 to 43.0%). The regression of the residuals of TL on SVL on the number of subcaudal scales was not significant (r^2^ = 0.073, F_1,33_ = 2.59, *p* = 0.12, n = 35). Similarly, the regression of the residuals of TL on SVL on the length of the hemipenes pocket was not significant (r^2^ = 0.072, F_1,33_ = 2.59, *p* = 0.12, n = 35). In addition, the regression of the residuals of the length of the subcaudal cavity on SVL on the residuals of TL on SVL was not significant (r^2^ = 0.086, F_1,33_ = 3.12, *p* = 0.09, n = 35). Consequently, and similar to earlier findings of King^5^, the morphological constraint hypothesis within species was not supported using these calculations.

**Figure 2:**
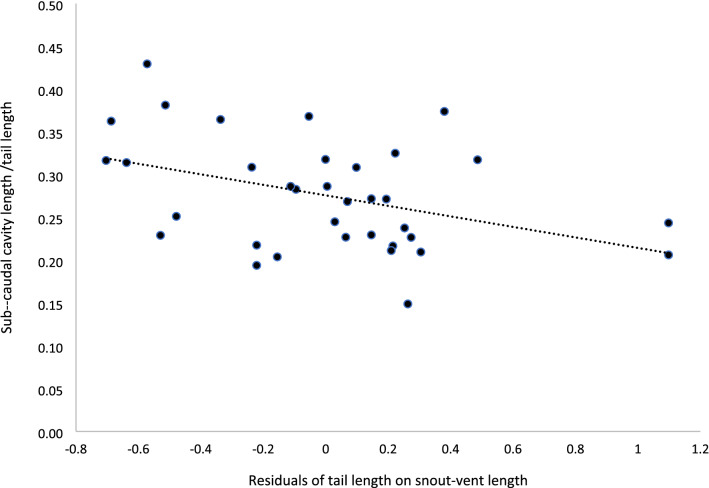
The effect of the residuals of tail length on snout-vent length, on the ratio of the subcaudal cavity length and tail length. Thus, the reproductive structure occupies a greater proportion of the tail in individuals with relatively shorter tails conforming to the intraspecific morphological constraint hypothesis^5^. See text for statistical tests.

King^5^ stated that the male reproductive structure “should occupy a greater proportion of male TL in short-tailed individuals than in long-tailed individuals”. Interestingly, the regression of the ratio of the hemipenes pocket length and TL on the residuals of TL on SVL was significant and was correlated negatively, as expected from King’s prediction (r^2^ = 0.164, F_1,33_ = 6.46, *p* = 0.016, n = 35; slope = −0.061 ± 0.024; Fig. 2). Therefore, the reproductive structure in individuals with relatively shorter tails occupy a greater proportion of the TL than in individuals with relatively longer tails, which supports the morphological constraint hypothesis (Prediction 1B^5^). Yet, the morphological constraint hypothesis does not consider overall TL in males but rather the difference between sexes. This premise was further emphasized by King^5^ when he stated that the hypothesis addresses “the evolution of differences in tail length between males and females and not the evolution of tail length per se”. However, it seems that both King^5^ and Shine et al.^11^ missed that important point in their intraspecific analyses.

Our novel approach tested the morphological constraint hypothesis through the difference between sexes. This could be achieved by statistically removing the effect in males in comparison with females. The average TL:SVL ratio after removing the length of the subcaudal cavity [(TL – length of the hemipenes pocket):SVL ratio] in males was 0.0948 ± 0.0033 which was similar to the TL:SVL ratio in females (0. 0910; t_144_ = 0.944, df = 144, *p* = 0.347). Therefore, the difference between the sexes in average TL:SVL or in the slopes of the regressions of TL on SVL was due mainly to the length of the hemipenes pocket which surrounds the hemipenes. This finding strongly supports the morphological constraint hypothesis and provides an explanation to the sexual difference in TL, at least in *Cerastes vipera*.

## Conclusions

The relatively longer tail in *C. vipera* males than in females is explained fully by the length of the hemipenes pocket. In addition, the relatively short tail of *C. vipera* presents a constraint to the reproductive structures in males, as the length of the hemipenes pocket occupies a greater proportion in shorter- than in longer-tailed individuals. This is the first report presenting these intraspecific findings in support of the morphological constraint hypothesis. Whether these findings are widespread among snake families, within Viperidae, or specifically within *C. vipera* warrants further studies. It should be noted that for such assessments, relative and not absolute measures should be taken for each sex, sample size of the population should be sufficiently large to capture actual variance within the population as already mentioned by Klauber^2^, and analysis should not be limited to proportions but should also include regressions and/or analysis of covariance. In addition, it is important to clarify that the use of ratios such as tail:SVL that were common in earlier publications (i.e., Klauber^2^) was calculated in the present study to allow comparisons with the earlier studies, but the use of simple regression analyses to indicate comparative trends (i.e., King^5^) is not recommended. ANCOVA models as used in the current study and also employed by Maritz^20^ is a preferred statistical approach for comparative studies.

## Supplementary Information

Supplementary Information 1.

Supplementary Information 2.

## Data Availability

All data used in this study are included in the manuscript and in Appendices 1 and 2.

## References

[1] Clark DR (1966). Notes on sexual dimorphism in tail-length in American snakes. Trans. Kans. Acad. Sci..

[2] Klauber LM (1943). Tail-length differences in snakes with notes on sexual dimorphism and the coefficient of divergence. Bull. Zool. Soc. San. Diego..

[3] Tomović L, Radojičić J, Džukić G, Kalezic M (2002). Sexual dimorphism of the Sand Viper (*Vipera ammodytes* L.) from the central part of Balkan Peninsula. Russian J. Herpetol..

[4] Tomović LM, Crnobrnja-Isailović JM, Ajtić RD, Aleksić ID, Djordjević SZ (2009). When do meadow vipers (*Vipera ursinii*) become sexually dimorphic? Ontogenetic patterns of sexual size dimorphisms. J. Zool. Syst. Evolut. Res..

[5] King RB (1989). Sexual dimorphism in snake tail length: sexual selection, natural selection, or morphological constraint?. Biol. J. Linn. Soc..

[6] Kaufman GA, Gibbons JW (1975). Weight-length relationships in thirteen species of snakes in the southeastern United States. Herpetologica.

[7] Semlitsch RD, Gibbons JW (1982). Body size dimorphism and sexual selection in two species of water snakes. Copeia.

[8] Darwin C (1859). On the Origin of Species by Means of Natural Selection.

[9] Zahavi, A. & Zahavi, A. The Handicap Principle: A Missing Piece of Darwin’s Puzzle (Oxford University Press, 1997).

[10] Sivan J, Hadad S, Tesler I, Rosenstrauch A, Degen AA, Kam M (2020). Relative tail length relates to body condition in males but not in females in the sexually dimorphic crowned leafnose (*Lytorhynchus diadema*). Sci. Rep..

[11] Shine R, Olsson MM, Moore IT, LeMaster MP, Mason RT (1999). Why do male snakes have longer tails than females?. Proc. R. Soc. B.

[12] Sivan J, Kam M, Hadad S, Degen AA, Rozenboim I, Rosenstrauch A (2013). Temporal activity and dietary selection in two coexisting desert snakes, the Saharan sand viper (*Cerastes vipera*) and the crowned leafnose (*Lytorhynchus diadema*). Zoology.

[13] Tesler I, Sivan J, Rosenstrauch A, Horesh SJA, Degen AA, Kam M (2019). Sexual dichromatisation and sexual differences in hunting behavior and dietary intake in a free-ranging small viperid snake. Cerastes vipera. Behav. Proc..

[14] Horesh, S. J. A. *et al.* Seasonal biotic and abiotic factors affecting hunting strategy in free-living Saharan sand vipers, *Cerastes vipera*. * Behav. Proc*. **135**, 40–44 (2017). 10.1016/j.beproc.2016.11.013 27899311

[15] Marx H (1958). Sexual dimorphism in coloration in the viper *Cerastes vipera* L. Nat. Hist. Miscellanea CAS..

[16] Sivan J, Kam M, Hadad S, Degen AA, Rosenstrauch A (2015). Body size and seasonal body condition in two small coexisting desert snake species, the Saharan sand viper (*Cerastes vipera*) and the crowned leafnose (*Lytorhynchus diadema*). J. Arid Environ..

[17] Bonnet X, Lorioux S, Pearson D, Aubret F, Bradshaw D, Delmas V, Fauvel T (2011). Which proximate factor determines sexual size dimorphism in tiger snakes?. Biol. J. Linn. Soc..

[18] Sivan J, Kam M, Hadad S, Degen AA, Rozenboim I, Rosenstrauch A (2012). Reproductive cycle of free-living male Saharan sand vipers, Cerastes vipera (Viperidae) in the Negev desert, Israel. Gen. Comput. Endocrinol..

[19] Baum CF (2008). Modeling proportions. Stat. J..

[20] Maritz B, Alexander GJ (2011). Morphology, sexual dimorphism, and growth in the smallest viperid, *Bitis schneideri* (Reptilia: Squamata: Viperidae). J. Herpetol..

[21] Lillywhite, H.B., De Delva, P. & Noonan, B.P. Patterns of gut passage time and the chronic retention of fecal mass in viperid snakes. In: Biology of the Vipers (eds Schuett, G., Hӧggren, W.M., Douglas, M.E. & Greene, H.W.) (Eagle Mountain Publishing USA, pp. 497–506, 2002).

