# Supplementary material for: Testing the morphological constraint hypothesis of tail length in the sexually dimorphic Cerastes vipera and new perspectives
Source: Sci Rep. 2023 Mar 17;13:4447. doi: 10.1038/s41598-023-31624-6 (PMC10023687; doi:10.1038/s41598-023-31624-6)
Supplement: Supplementary file 1 — Supplementary Information 1. [file 41598_2023_31624_MOESM1_ESM.pdf]

Appendix 1.

Snout-vent length (SVL; cm), tail length (TL; cm), and the residuals of TL on SVL in free-ranging *Cerastes vipera* males (M) and females (F).
See text for details.

Sex	SVL	TL	Residuals
F	15.3	1.2	-0.4533
F	15.5	1.5	-0.1753
F	15.7	1.3	-0.3972
F	16.2	1.5	-0.2521
F	16.5	1	-0.7851
F	16.5	1	-0.7851
F	16.5	1	-0.7851
F	16.5	1	-0.7851
F	16.5	1.7	-0.0851
F	16.8	1.7	-0.1180
F	17	1.5	-0.3400
F	17	2	0.1600
F	17.1	1.5	-0.3509
F	17.3	1.5	-0.3729
F	17.5	1.5	-0.3949
F	17.5	1.7	-0.1949
F	17.5	2	0.1051
F	17.5	2	0.1051
F	17.6	1.9	-0.0058
F	17.6	1.9	-0.0058
F	17.7	1.5	-0.4168
F	17.9	1.3	-0.6388
F	18	1	-0.9498
F	18	1.2	-0.7498
F	18	1.5	-0.4498
F	18.1	1.9	-0.0607
F	18.3	1.7	-0.2827
F	18.3	2	0.0173
F	18.4	1.6	-0.3937
F	18.4	2.5	0.5063
F	18.7	1.8	-0.2266
F	18.7	1.8	-0.2266
F	18.7	1.8	-0.2266
F	18.8	1.7	-0.3376
F	18.8	1.7	-0.3376
F	18.9	1.6	-0.4486
F	18.9	1.8	-0.2486
F	19	2	-0.0596
F	19.2	2	-0.0815
F	19.4	1.6	-0.5035

F	19.4	2.6	0.4965
F	19.5	1.5	-0.6145
F	19.5	2	-0.1145
F	19.6	1.9	-0.2254
F	19.9	1.7	-0.4584
F	20	1.5	-0.6694
F	20.2	1.8	-0.3913
F	20.2	2.8	0.6087
F	20.3	1.7	-0.5023
F	20.3	1.7	-0.5023
F	20.3	1.9	-0.3023
F	20.8	1.7	-0.5572
F	21.1	1.9	-0.3901
F	21.1	1.9	-0.3901
F	21.2	1.8	-0.5011
F	21.2	2.1	-0.2011
F	21.3	1.2	-1.1121
F	21.4	2.1	-0.2231
F	21.4	2.3	-0.0231
F	21.6	1.9	-0.4450
F	21.8	1.7	-0.6670
F	21.8	2	-0.3670
F	21.8	2.2	-0.1670
F	21.8	2.2	-0.1670
F	21.9	2.1	-0.2780
F	21.9	2.1	-0.2780
F	22	2	-0.3889
F	22	2	-0.3889
F	22	2.1	-0.2889
F	22.2	1.8	-0.6109
F	22.2	2.3	-0.1109
F	22.3	1.8	-0.6219
F	22.5	1.5	-0.9438
F	22.5	2	-0.4438
F	22.5	2	-0.4438
F	22.5	2	-0.4438
F	22.5	2	-0.4438
F	22.5	2.5	0.0562
F	22.5	2.5	0.0562
F	22.6	2.2	-0.2548
F	22.6	2.4	-0.0548
F	22.9	2.1	-0.3878
F	22.9	2.1	-0.3878
F	23	2	-0.4987
F	23	2	-0.4987
F	23	2.5	0.0013
F	23.13	2.2	-0.3130

F	23.2	1.8	-0.7207
F	23.2	2.6	0.0793
F	23.4	2.2	-0.3427
F	23.5	1.9	-0.6536
F	23.7	2	-0.5756
F	23.7	2.3	-0.2756
F	23.7	2.3	-0.2756
F	23.7	2.3	-0.2756
F	24	2	-0.6085
F	24	2	-0.6085
F	24.2	1.8	-0.8305
F	24.3	2.2	-0.4415
F	24.8	2.2	-0.4964
F	24.9	2.1	-0.6074
F	24.9	2.5	-0.2074
F	25	1.5	-1.2183
F	25.4	2.1	-0.6623
F	25.5	1.5	-1.2732
F	25.5	2.2	-0.5732
F	25.5	2.7	-0.0732
F	25.7	2.3	-0.4952
F	25.9	2.1	-0.7172
F	26.5	2.5	-0.3830
F	27.3	2.7	-0.2709
M	15	1.5	-0.1204
M	15	2	0.3796
M	15.1	1.9	0.2687
M	15.2	1.8	0.1577
M	15.2	1.3	-0.3423
M	15.5	1.8	0.1247
M	15.5	2	0.3247
M	15.9	1.6	-0.1192
M	16	1.5	-0.2302
M	16	1.5	-0.2302
M	16	2	0.2698
M	16	1.5	-0.2302
M	16.1	1.7	-0.0411
M	16.1	1.9	0.1589
M	16.1	1.9	0.1589
M	16.2	2	0.2479
M	16.5	1.5	-0.2851
M	16.5	2	0.2149
M	16.6	2.4	0.6040
M	16.6	1.9	0.1040
M	16.6	3	1.2040
M	16.8	2.2	0.3820
M	16.8	2.2	0.3820

M	16.9	2.1	0.2710
M	16.9	2.1	0.2710
M	17	1.5	-0.3400
M	17	1.5	-0.3400
M	17	2	0.1600
M	17	2	0.1600
M	17	2	0.1600
M	17	2	0.1600
M	17	2	0.1600
M	17	2	0.1600
M	17	2.1	0.2600
M	17.1	1.8	-0.0509
M	17.2	2.3	0.4381
M	17.3	2	0.1271
M	17.5	2	0.1051
M	17.9	2.1	0.1612
M	17.9	2.6	0.6612
M	18	1.5	-0.4498
M	18	1.5	-0.4498
M	18	2	0.0502
M	18	2.5	0.5502
M	18.1	2.9	0.9393
M	18.2	1.8	-0.1717
M	18.2	2	0.0283
M	18.2	2.3	0.3283
M	18.7	1.8	-0.2266
M	18.7	2.3	0.2734
M	18.7	1.8	-0.2266
M	18.7	2.5	0.4734
M	18.7	2.3	0.2734
M	18.8	1.7	-0.3376
M	18.9	2.1	0.0514
M	19	2.5	0.4404
M	19.1	2.2	0.1295
M	19.1	2.4	0.3295
M	19.1	2.4	0.3295
M	19.1	2.4	0.3295
M	19.1	2.4	0.3295
M	19.1	2.9	0.8295
M	19.2	2.3	0.2185
M	19.4	1.8	-0.3035
M	19.5	2.5	0.3855
M	19.5	2.5	0.3855
M	19.5	2.5	0.3855
M	19.5	2.5	0.3855
M	19.6	2.4	0.2746
M	19.7	2.5	0.3636

M	19.8	2.7	0.5526
M	19.8	2.2	0.0526
M	19.8	3.2	1.0526
M	19.9	1.6	-0.5584
M	19.9	2.6	0.4416
M	20	2.5	0.3306
M	20	2.5	0.3306
M	20	2.5	0.3306
M	20.1	2.1	-0.0803
M	20.1	1.9	-0.2803
M	20.1	2.4	0.2197
M	20.1	2.4	0.2197
M	20.1	2.9	0.7197
M	20.2	2.8	0.6087
M	20.2	2.5	0.3087
M	20.2	2.8	0.6087
M	20.3	2.7	0.4977
M	20.35	2.2	-0.0078
M	20.4	2.6	0.3867
M	20.4	2.6	0.3867
M	20.4	2.9	0.6867
M	20.5	2.5	0.2757
M	20.5	2.5	0.2757
M	20.5	2.5	0.2757
M	20.5	2.5	0.2757
M	20.5	3	0.7757
M	20.6	2.8	0.5648
M	20.6	2.4	0.1648
M	20.6	2.9	0.6648
M	20.6	2.9	0.6648
M	20.7	2.3	0.0538
M	20.7	2.8	0.5538
M	21	2	-0.2791
M	21	2.5	0.2209
M	21	3	0.7209
M	21.1	2.9	0.6099
M	21.1	2.9	0.6099
M	21.2	2.8	0.4989
M	21.4	2.6	0.2769
M	21.4	2.6	0.2769
M	21.4	2.1	-0.2231
M	21.4	2.6	0.2769
M	21.5	2	-0.3340
M	21.5	2	-0.3340
M	21.5	2.5	0.1660
M	21.5	3	0.6660
M	21.5	3	0.6660

M	21.5	3	0.6660
M	21.6	3.1	0.7550
M	21.6	2.7	0.3550
M	21.7	2.8	0.4440
M	21.8	2.7	0.3330
M	21.8	2.7	0.3330
M	21.9	2.3	-0.0780
M	21.9	3.1	0.7220
M	22	2.5	0.1111
M	22	2.5	0.1111
M	22	2.8	0.4111
M	22	3	0.6111
M	22	3	0.6111
M	22	3	0.6111
M	22.1	2.9	0.5001
M	22.2	2.8	0.3891
M	22.2	2.8	0.3891
M	22.2	2.8	0.3891
M	22.2	2.8	0.3891
M	22.2	3.3	0.8891
M	22.3	2.7	0.2781
M	22.3	3.2	0.7781
M	22.4	1.6	-0.8329
M	22.4	2.6	0.1671
M	22.4	2.8	0.3671
M	22.5	2.5	0.0562
M	22.5	3	0.5562
M	22.5	3.2	0.7562
M	22.6	2.9	0.4452
M	22.8	1.9	-0.5768
M	22.8	3.2	0.7232
M	22.8	3.2	0.7232
M	23	2.5	0.0013
M	23	2.8	0.3013
M	23	3	0.5013
M	23	3	0.5013
M	23.1	3.1	0.5903
M	23.4	3.1	0.5573
M	23.5	3	0.4464
M	23.7	3.3	0.7244
M	23.8	3.2	0.6134
M	24	2	-0.6085
M	24	3	0.3915
M	24.2	2.5	-0.1305
M	24.2	3.3	0.6695
M	24.3	2.2	-0.4415
M	24.4	3.1	0.4475

M	24.5	3	0.3366
M	25.2	3.1	0.3597
