# Supplementary material for: Testing the morphological constraint hypothesis of tail length in the sexually dimorphic Cerastes vipera and new perspectives
Source: Sci Rep. 2023 Mar 17;13:4447. doi: 10.1038/s41598-023-31624-6 (PMC10023687; doi:10.1038/s41598-023-31624-6)
Supplement: Supplementary file 2 — Supplementary Information 2. [file 41598_2023_31624_MOESM2_ESM.pdf]

Appendix 2.

Body mass (mb), snout-vent length (SVL; cm), tail length (TL; cm),
number of sub-caudal scales (SC) and the length of the hemipenes pocket (HP; cm)
in *Cerastes vipera* males. See text for details.

Specimen #	mb	SVL	TL	SC	HP
1	14.86	21.5	2.70	7	0.764
3	17.05	21.5	2.80	7	0.802
4	14.20	20.7	2.80	7	0.864
5	20.44	22.4	2.90	7	0.922
6	17.26	23.3	3.20	8	0.869
7	12.91	20.8	3.20	9	1.016
8	18.33	20.7	3.80	7	0.784
9	20.32	21.3	2.20	8	0.945
10	17.24	20.8	2.20	5	0.839
11	18.10	19.7	2.80	8	0.607
12	16.36	22.2	3.10	8	1.008
13	16.08	21.0	2.40	7	0.875
14	13.49	20.6	2.90	4	0.613
15	18.34	21.0	3.00	5	0.447
16	15.32	22.0	3.00	8	0.689
17	11.40	18.3	2.20	5	0.427
18	18.41	23.0	3.00	6	0.735
19	13.78	21.5	3.10	6	0.650
20	12.19	23.5	2.50	6	0.573
23	19.41	26.7	2.70	7	0.855
24	14.99	20.0	3.00	9	1.122
25	14.01	19.2	2.80	6	0.635
27	19.79	20.7	3.80	8	0.926
28	15.97	22.8	3.20	6	0.761
29	16.02	21.8	2.90	6	0.779
30	17.27	22.0	2.80	6	1.031
31	15.33	21.0	2.50	8	0.772
33	13.10	20.3	2.50	5	0.510
34	16.60	22.0	3.00	8	0.816
36	8.13	16.3	1.50	6	0.544
38	11.25	18.3	2.20	7	0.479
39	15.21	22.7	3.00	8	0.681
40	10.25	19.3	1.90	6	0.597
41	20.24	22.5	2.80	6	0.802
42	13.28	20.5	2.20	7	0.553
